# Supplementary material for: Analysis of PPARγ Signaling Activity in Psoriasis
Source: Int J Mol Sci. 2021 Aug 10;22(16):8603. doi: 10.3390/ijms22168603 (PMC8395241; doi:10.3390/ijms22168603)
Supplement: Supplementary file 1 [file ijms-22-08603-s001.zip › Supplemental materials_Analysis of PPARg signaling activity in psoriasis/Pathway models/Models images and html files/Interleukin-17 and interleukin-22 signaling in psoriasis/4461.html]

FLG


# Protein FLG

|  |  |
| --- | --- |
| URN | urn:agi-llid:2312 |
| Total Entities | 0 |
| Connectivity | 964 |
| Name | FLG |
| Description | filaggrin |
| Notes | The protein encoded by this gene is an intermediate filament-associated protein that aggregates keratin intermediate filaments in mammalian epidermis. It is initially synthesized as a polyprotein precursor, profilaggrin (consisting of multiple filaggrin units of 324 aa each), which is localized in keratohyalin granules, and is subsequently proteolytically processed into individual functional filaggrin molecules. Mutations in this gene are associated with ichthyosis vulgaris.[provided by RefSeq, Dec 2009] |
| Primary Cell Localization | Cytoplasm |

---

|  |  |
| --- | --- |
| Pathway | Epidermal Barrier Dysfunction in Atopic Dermatitis |
|  | Genes with Mutations Associated with Atopic Dermatitis |
|  | Proteins with Altered Expression in Atopic Dermatitis |
|  | Proteins Involved in Atopic Dermatitis |
|  | Atopic Dermatitis |
|  | Proteins Involved in Ichthyosis |
|  | Ichthyosis Vulgaris |
|  | Genes with Mutations Associated with Ichthyosis |
|  | Proteins with Altered Expression in Ichthyosis |
|  | IL22 Induces Keratinocyte Proliferation in Psoriasis |
|  | Proteins with Altered Expression in Psoriasis |
|  | Elevated Receptors -> Expression Targets in Skin |
|  | Dioxin Induced Chloracne (Hypothesis) |
|  | Overview. Diseases of the skin (chapter 11) |
|  | 2\_Acute phase initiation in atopic dermatitis |
|  | 2\_Interleukin-17 and interleukin-22 signaling in psoriasis |
|  | 1\_Ichthyosis vulgaris overview |
|  | Interleukin-17 and interleukin-22 signaling in psoriasis |

---

|  |  |
| --- | --- |
| Group | Genes with Mutations Associated with Atopic Dermatitis |
|  | Genes with Mutations Associated with Psoriasis |

---

|  |  |
| --- | --- |
| MedScan ID | 2312 |

---

|  |  |
| --- | --- |
| LocusLink ID | 2312 |

---

|  |  |
| --- | --- |
| Alias | fillaggrins |
|  | flaky tail protein |
|  | profilaggrin |
|  | filaggrin |
|  | profillaggrin |
|  | fillagrin |
|  | LOC400786 |
|  | OTTHUMP00000014057 |
|  | flaky tail mutant |
|  | Pflg |
|  | Filaggrin (profilaggrin) |
|  | ichthyosis vulgaris gene |
|  | epidermal filaggrin |
|  | LOC619541 |
|  | Dermatitis, atopic, II |
|  | Dermatitis, atopic, 2 |
|  | ATOD2 |
|  | filaggrins |
|  | proFLG |

---

|  |  |
| --- | --- |
| GO ID | 0005509 |
|  | 0030280 |
|  | 0005198 |
|  | 0046914 |
|  | 0070268 |
|  | 0061436 |
|  | 0030216 |
|  | 0007275 |
|  | 0018149 |
|  | 0062023 |
|  | 0001533 |
|  | 0005829 |
|  | 0005882 |
|  | 0043231 |
|  | 0036457 |
|  | 0005634 |
|  | 0016023 |

---

|  |  |
| --- | --- |
| KEGG ID | hsa:2312 |

---

|  |  |
| --- | --- |
| Organism | Homo sapiens {Organism urn:agi-taxid:9606} |
|  | Homo sapiens |

---

|  |  |
| --- | --- |
| OMIM ID | 135940 |
|  | 605803 |
|  | 146700 |

---

|  |  |
| --- | --- |
| Hugo ID | 3748 |
|  | HGNC:3748 |

---

|  |  |
| --- | --- |
| Human chromosome position | 1q21.3 |

---

|  |  |
| --- | --- |
| Swiss-Prot Accession | P20930 |
|  | P20930.3 |
|  | Q01720 |
|  | Q5T583 |
|  | Q9UC71 |

---

|  |  |
| --- | --- |
| PIR ID | A32947 |
|  | A45135 |
|  | A48118 |

---

|  |  |
| --- | --- |
| GenBank ID | NC\_000001 |
|  | NM\_002016 |
|  | NP\_002007 |
|  | NG\_016190 |
|  | AF043380 |
|  | AAC23559 |
|  | AH002947 |
|  | AAA60177 |
|  | AH003056 |
|  | AAA63243 |
|  | AL356504 |
|  | CH471121 |
|  | EAW53383 |
|  | EAW53384 |
|  | EAW53385 |
|  | CS673440 |
|  | CAP07487 |
|  | CS690823 |
|  | CAP11455 |
|  | FB665576 |
|  | CAR97069 |
|  | GM706788 |
|  | CAT82291 |
|  | HF583667 |
|  | CCQ43164 |
|  | JN184345 |
|  | AFH55054 |
|  | JN184346 |
|  | AFH55055 |
|  | JN184347 |
|  | AFH55056 |
|  | JN184348 |
|  | AFH55057 |
|  | JN184349 |
|  | AFH55058 |
|  | JN184350 |
|  | AFH55059 |
|  | M60494 |
|  | AAA63244 |
|  | M96943 |
|  | AAA36487 |
|  | AB208881 |
|  | BAD92118 |
|  | L01090 |
|  | AAA60176 |
|  | M24355 |
|  | AAA52454 |
|  | M60495 |
|  | AAA63245 |
|  | M60499 |
|  | AAA63246 |
|  | M60500 |
|  | AAA63247 |
|  | M60502 |
|  | AAA63248 |
|  | P20930 |
|  | XM\_011509329 |
|  | XP\_011507631 |
|  | NC\_018912 |
|  | AMYH02001891 |
|  | L01088 |
|  | L01089 |
|  | M60503 |
|  | GU014833 |
|  | ACX32320 |
|  | AC\_000133 |
|  | ABBA01049414 |

---

|  |  |
| --- | --- |
| Swiss-Prot ID | FILA\_HUMAN |

---

|  |  |
| --- | --- |
| Cell Localization | Cytoplasmic granule |

---

|  |  |
| --- | --- |
| Ensembl ID | ENSG00000143631 |
|  | ENSP00000357789.1 |
|  | ENST00000368799.1 |
|  | ENSP00000357789 |
|  | ENST00000368799 |

---

|  |  |
| --- | --- |
| Unigene ID | Hs.654510 |

---
